# Magnetic resonance imaging evaluation of spinal cord lesions: what can we find? - Part 2. Inflammatory and infectious injuries

**DOI:** 10.1590/0100-3984.2020.0128

**Published:** 2021

**Authors:** Ronaldo Gonçalves Pereira, Bruno Niemeyer de Freitas Ribeiro, Thais Ribeiro Gomes Coutinho Pereira, Paulo Roberto Valle Bahia, Edson Marchiori

**Affiliations:** 1 Hospital Casa de Portugal / 3D Diagnóstico por Imagem, Rio de Janeiro, RJ, Brazil.; 2 Grupo Labs Fleury/RJ, Rio de Janeiro, RJ, Brazil.; 3 Instituto Estadual do Cérebro Paulo Niemeyer, Rio de Janeiro, RJ, Brazil.; 4 Universidade Federal do Rio de Janeiro (UFRJ), Rio de Janeiro, RJ, Brazil.

**Keywords:** Magnetic resonance imaging, Spinal cord/pathology, Spinal cord diseases, Inflammation, Infection, Ressonância magnética, Medula espinal/patologia, Doenças da medula espinal, Inflamação, Infecção

## Abstract

Diseases involving the spinal cord include a heterogeneous group of abnormalities, including those of inflammatory, infectious, neoplastic, vascular, metabolic, and traumatic origin. Making the clinical differentiation between different entities is often difficult, magnetic resonance imaging being the diagnostic method of choice. Although the neuroimaging findings are not pathognomonic, many are quite suggestive, and the radiologist can assist in the diagnosis and, consequently, in the therapeutic guidance. In this second part of our article, the objective is to review the magnetic resonance imaging findings of the main inflammatory and infectious spinal cord injuries.

## Abstract (pt)

As doenças que envolvem a medula espinal incluem um grupo heterogêneo de anomalias, englobando causas inflamatórias, infecciosas, neoplásicas, vasculares, metabólicas e traumáticas. Muitas vezes a diferenciação clínica entre as diversas entidades é difícil, sendo a ressonância magnética a modalidade de escolha na investigação diagnóstica. Apesar de os achados de neuroimagem não serem patognomônicos, muitos são bastante sugestivos, podendo o radiologista auxiliar no diagnóstico e, consequentemente, na orientação terapêutica. O objetivo desta segunda parte do artigo é revisar os achados de ressonância magnética das principais lesões medulares inflamatórias e infecciosas.

## INTRODUCTION

The spinal cord is the portion of the central nervous system that is within the vertebral canal, extending from the foramen magnum to the conus medullaris at the L1/L2 level, being surrounded by cerebrospinal fluid and contained by the thecal sac. Countless diseases can affect this region, leading to motor, sensory, and autonomic alterations, and magnetic resonance imaging (MRI) findings are essential for diagnostic elucidation and therapeutic orientation. The evaluation of the nervous system by imaging methods has been the subject of a series of recent articles in the radiology literature of Brazil^**(1-5)**^. In this second part of our article, the objective is to review the MRI findings of the main inflammatory and infectious spinal cord injuries.

## INFLAMMATORY CAUSES

### Acute disseminated encephalomyelitis

**Figure 1:**
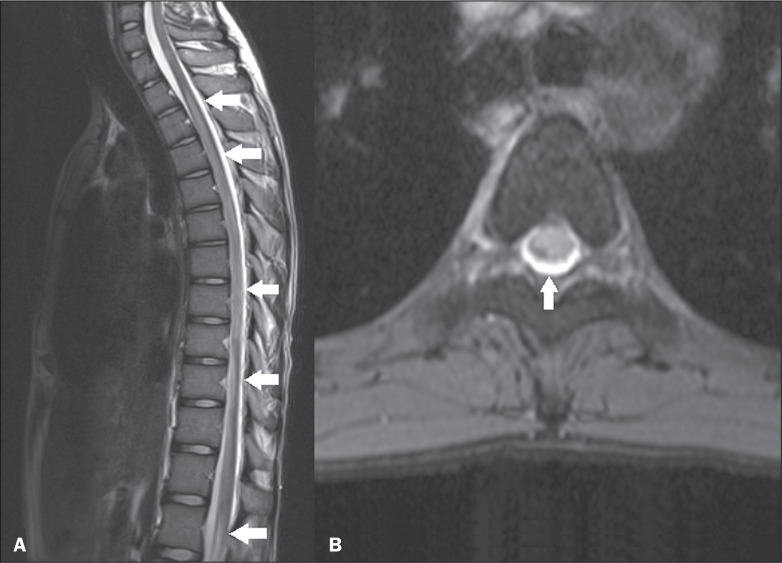
Acute disseminated encephalomyelitis. Sagittal T2-weighted and axial T2*-weighted MRI sequences (A and B, respectively) showing a longitudinally extensive hyperintense spinal cord lesion (arrows in A), affecting more than two-thirds of the sectional area of the spinal cord (arrow in B).

Acute disseminated encephalomyelitis is a demyelinating immune-mediated disease, classically of a monophasic course, that is typically secondary to a viral infection or vaccination, spinal cord involvement occurring in up to one third of cases. Spinal cord lesions are typically longitudinally extensive (continuous involvement of ≥ three vertebral levels) and confluent, potentially affecting more than two thirds of the sectional area of the spinal cord. On T2-weighted MRI scans, such lesions are typically hyperintense, with variable contrast enhancement (Figure 1). The condition may regress after treatment^**(6)**^.

### Multiple sclerosis

**Figure 2:**
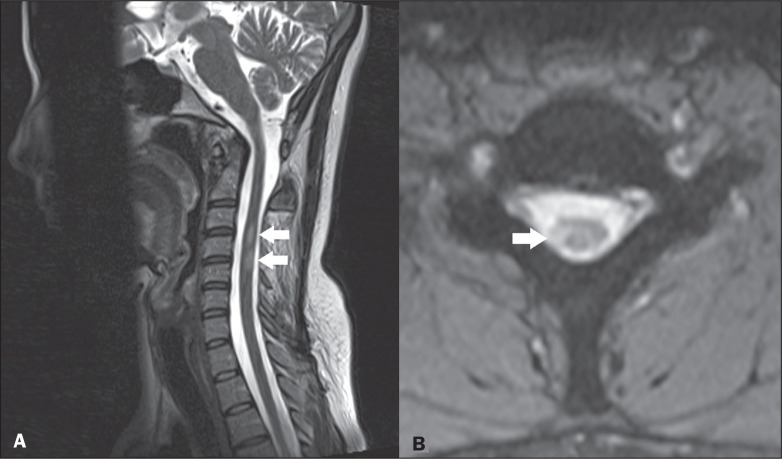
Multiple sclerosis. Sagittal T2-weighted and axial T2*-weighted MRI sequences (A and B, respectively) showing two small hyperintense lesions (arrows in A) affecting the right dorsolateral region (arrow in B).

Multiple sclerosis is a primary demyelinating disease that is more common in females and in the third and fourth decades of life. It is characterized by perivenular inflammation and demyelination with relative preservation of the axon. The most common site of involvement is the cervical spine. The lesions are typically well-defined and eccentric, primarily in a posterior location, affecting less than 50% of the sectional area of the spinal cord^**(6)**^. On T2-weighted MRI scans, the disease is characterized by hyperintense lesions and there can be contrast enhancement when there is disease activity (Figure 2). Spinal cord atrophy may occur and is common in the advanced stages.

### Neuromyelitis optica

**Figure 3:**
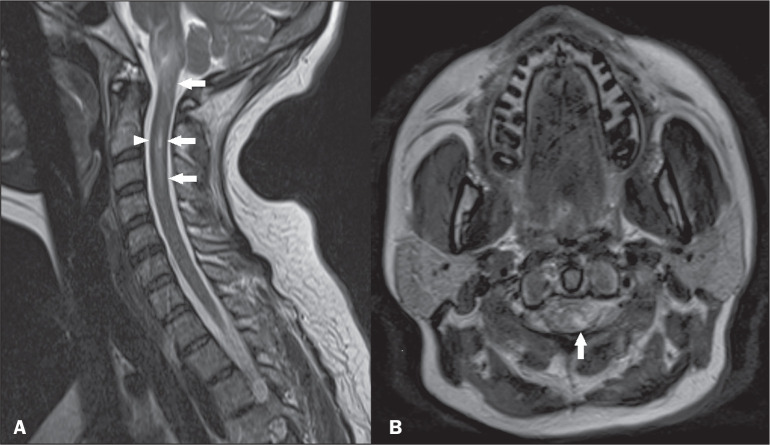
Neuromyelitis optica. Sagittal and axial T2-weighted MRI scans (A and B, respectively) showing a hyperintense spinal cord injury predominately in the central part of the spine (arrows in A and B), together with bright spotty lesions (arrowhead in A). There was also involvement extending to the area postrema (not shown).

Neuromyelitis optica is a demyelinating autoimmune disease induced by autoantibodies against aquaporin-4 water channels. The classic triad is optic neuritis, longitudinally extensive myelitis, and anti-aquaporin-4 antibody positivity^**(6)**^. On T2-weighted MRI scans, longitudinally extensive hyperintense lesions can be seen, primarily involving the cervical and thoracic spine, typically with concentric involvement around the central canal of the spine (Figure 3). A suggestive finding is the presence of bright spotty lesions (spinal cord foci with signal intensity higher than that of the cerebrospinal fluid on T2-weighted images). Spinal cord atrophy is common in the advanced stages.

### Acute transverse myelitis

Acute transverse myelitis is an immune-mediated inflammatory disorder associated with numerous infectious, inflammatory, and neoplastic diseases, rarely being idiopathic, affecting individuals of either gender and of any age. Clinically, it is characterized by acute symptoms. Paresis, ataxia, and sphincter dysfunction are common. On T2-weighted MRI, a longitudinally extensive hyperintense lesion is typically seen, primarily involving the cervical and thoracic spine, affecting more than two thirds of the sectional area. When there is contrast enhancement can occur, it is typically peripheral and irregular^**(7)**^.

### Anti-myelin oligodendrocyte glycoprotein antibody-associated myelitis

Anti-myelin oligodendrocyte glycoprotein (MOG) antibody-associated myelitis is a demyelinating inflammatory disorder associated with the presence of the immunoglobulin G antibody against MOG, being most common in the third decade of life, with a predilection for females. The most common presentation is a longitudinally extensive lesion, although small concentric hyperintense lesions can be seen on T2-weighted images and there can be isolated involvement of the conus medullaris, the latter being high suggestive of the diagnosis. Heterogeneous enhancement is common in most cases^**(8)**^.

### Lupus

**Figure 4:**
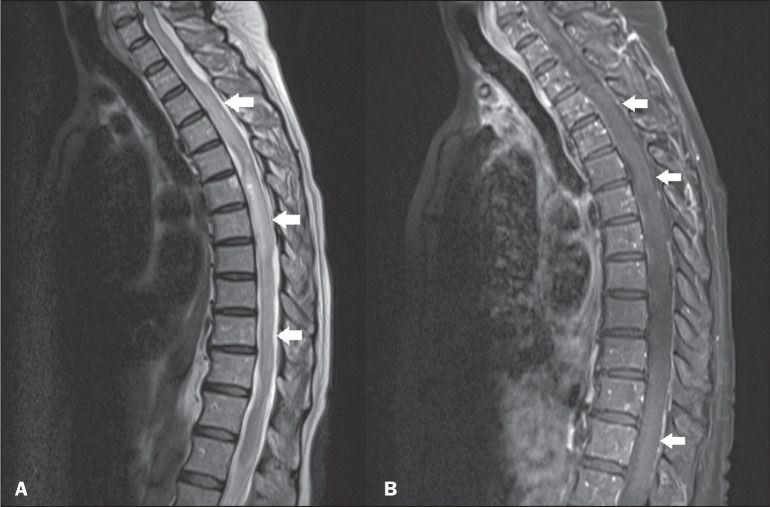
Lupus. Contrast-enhanced sagittal T2-weighted and T1-weighted MRI scans (A and B, respectively) showing a longitudinally extensive spinal cord lesion (arrows in A) in the thoracic spine, with slight contrast enhancement (arrows in B).

Myelopathy is a rare manifestation in lupus, affecting ≤ 2% of all lupus patients, and is typically associated with thrombosis and vasculitis. The thoracic spine is the most common site of involvement, where it manifests as a longitudinally extensive lesion occupying more than two thirds of the cross-sectional area. On MRI, the signal is isointense or hyperintense on T1-weighted images, hyperintense on T2-weighted images and shows variable contrast enhancement^**(9)**^, as illustrated in Figure 4.

## INFECTIOUS CAUSES

### Zika

**Figure 5:**
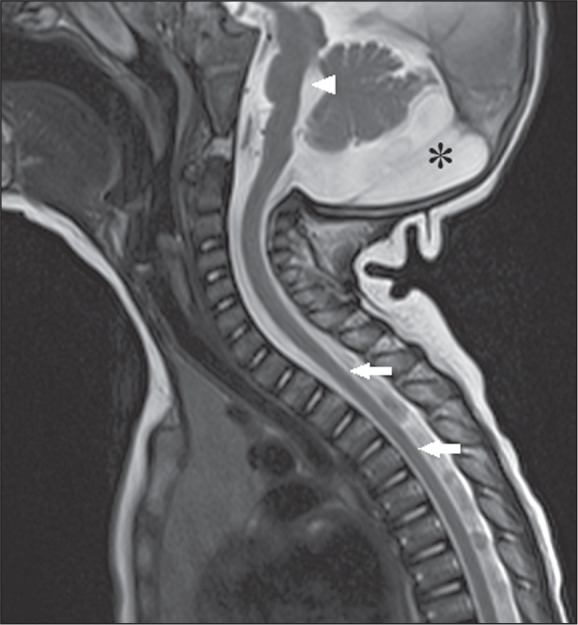
Zika. Sagittal T1-weighted MRI sequence showing narrowing of the thoracic spine (arrows) in a patient with congenital Zika syndrome, without arthrogryposis. Pontine hypoplasia (arrowhead) and an enlarged magna cistern (asterisk) are also shown.

In cases of Zika, the severity of changes in the spinal cord and nerve roots shows an apparent correlation with the presence of arthrogryposis. In such cases, MRI provides better visualization of spinal cord atrophy, as well as of the reduction of the anterior roots of the conus medullaris (Figure 5). However, in cases of Zika without arthrogryposis, only a reduction of spinal thickness in the thoracic region, discreet thinning of the anterior roots of the conus medullaris, and involvement of the anterior descending tracts are observed, with apparent preservation of the ascending posterior tracts^**(10)**^.

### Spinal cord schistosomiasis

**Figure 6:**
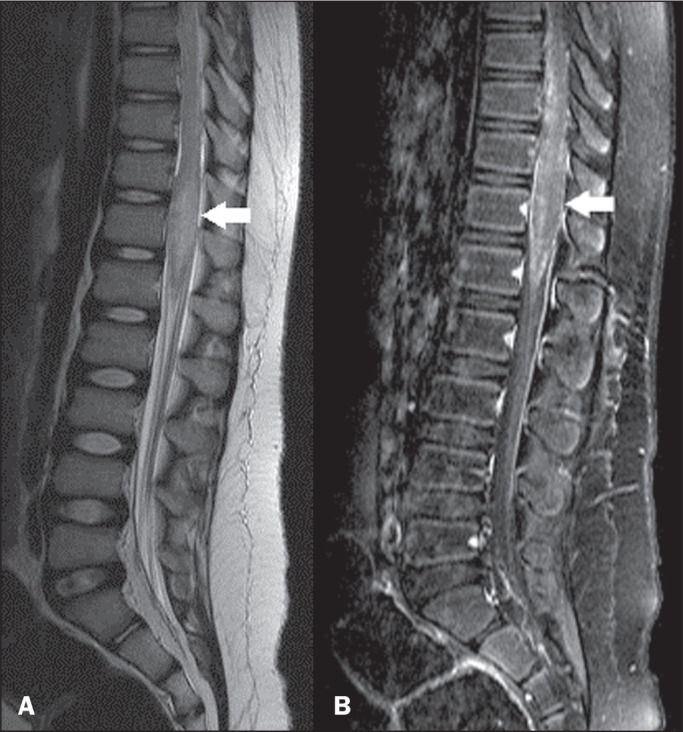
Spinal cord schistosomiasis. Sagittal T2-weighted MRI sequence (A) showing a central area of increased hyperintensity with poorly defined borders, together with an increase in the volume of the conus medullaris (arrow), resulting in obliteration of the corresponding anterior and posterior cerebrospinal space. Contrast-enhanced T1-weighted sequence (B) showing a heterogeneous tree-in-bud enhancement pattern (arrow). Courtesy of Dr. Gustavo Balthazar.

Spinal cord schistosomiasis is the most common ectopic form of infectious spinal cord injury, being the leading cause of nontraumatic non-neoplastic myelitis in endemic areas. The clinical picture is one of acute/subacute myelopathy. On MRI, spinal cord schistosomiasis typically presents as conus medullaris expansion, with a signal that is hypointense on T1-weighted images and hyperintense on T2-weighted images, with contrast media uptake (Figure 6), allowing the differential diagnosis to be made with anti-MOG antibody-associated demyelinating disease. The characteristic pattern of neuroschistosomiasis is linear, nodular tree-in-bud enhancement^**(11)**^.

### Human T-lymphotropic virus-1-associated myelopathy

**Figure 7:**
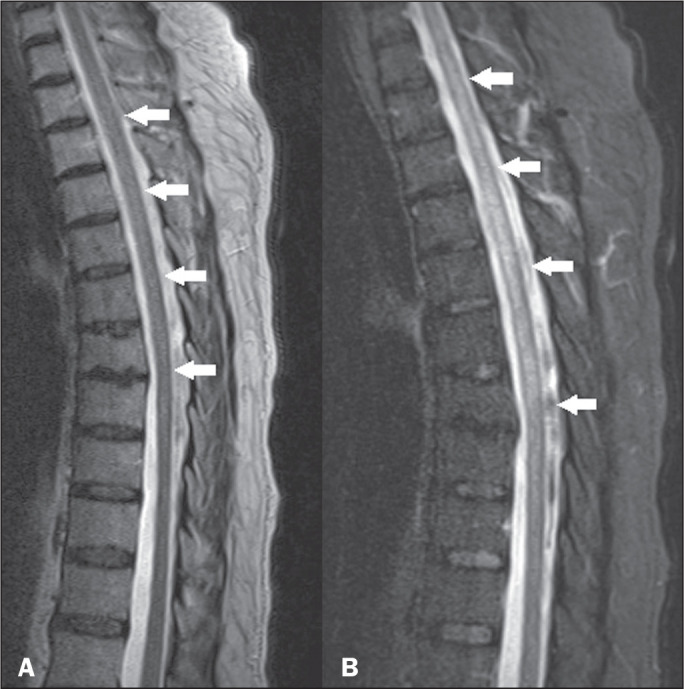
HTLV-1-associated myelopathy. Sagittal T2-weighted and STIR MRI sequences (A and B, respectively) showing a longitudinally extensive hyperintensity in the thoracic spine, together with mild atrophy (arrows).

Human T-lymphotropic virus-1 (HTLV-1)-associated myelopathy is a progressive chronic demyelinating disease affecting the spinal cord through multifactorial responses to HTLV-1 infection, being most common in females in the fourth decade of life. The most common clinical manifestation is chronic, slowly progressing spastic paraparesis^**(12)**^. On MRI, spinal cord atrophy and a hyperintense signal are seen in T2-weighted and short-tau inversion-recovery (STIR) sequences, more pronounced in the lateral columns and mainly involving the white matter, as well as the gray matter and anterior nerve roots (Figure 7). In cases of acute exacerbation, which is rare, there is spinal cord edema and peripheral contrast enhancement.

### AIDS-associated myelopathy

**Figure 8:**
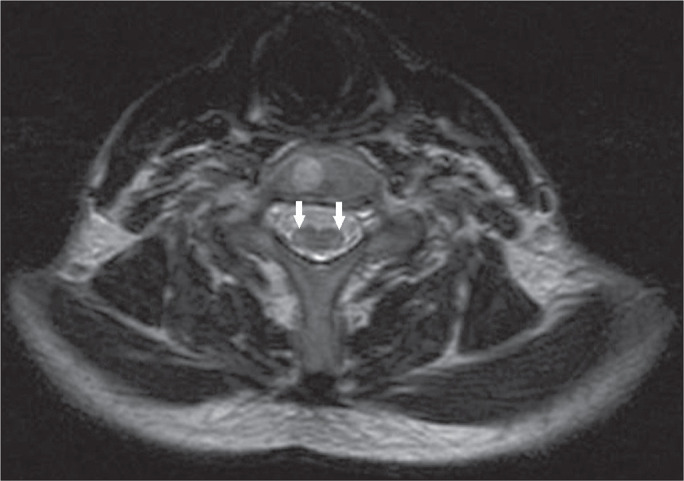
AIDS-associated myelopathy. Axial T2-weighted MRI sequence showing a hyperintense signal in the posterolateral regions (arrows). There was also spinal atrophy (not shown).

Typically, AIDS-associated myelopathy occurs in the final stages of the disease, being secondary to vacuolization of the white matter in the posterior and lateral regions of the spine, primarily in the thoracic spine. Clinically, AIDS-associated myelopathy is characterized by slowly progressing weakness in the lower limbs, gait disorders, sensory abnormalities, and impotence. On MRI, spinal cord atrophy is the most common finding, the most common site of involvement being the thoracic spine, followed by the cervical spine, T2-weighted images showing a well-defined symmetrical area of hyperintensity in the posterolateral region of the spine (Figure 8), predominantly in the gracile tract^**(13)**^.

### Poliomyelitis-like syndrome

**Figure 9:**
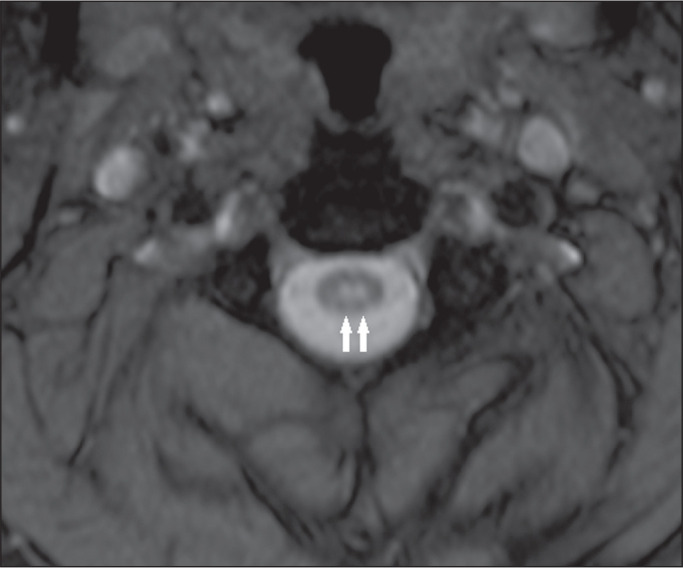
Poliomyelitis-like syndrome in a 5-year-old male. Axial T2-weighted MRI sequence showing a bilateral, symmetric hyperintense signal in the gray matter of the anterior horns of the spinal cord.

Poliomyelitis-like syndrome is defined as acute flaccid myelitis (inflammation of the spinal cord), which usually occurs after a viral disease (commonly related to enterovirus), characterized by flaccid paralysis, back pain, decreased sensitivity, and cranial nerve dysfunction. The MRI findings may be normal in the first 72 h and, when altered, typically indicate a longitudinally extensive lesion, primarily affecting the cervical and thoracic columns, characterized by a hyperintense signal on T2-weighted images, affecting the gray matter in the acute phase and the anterior horns of the spinal cord in the subacute phase (Figure 9). Enhancement of the cauda equina and cranial nerves may occur^**(14)**^.

**Figure 10:**
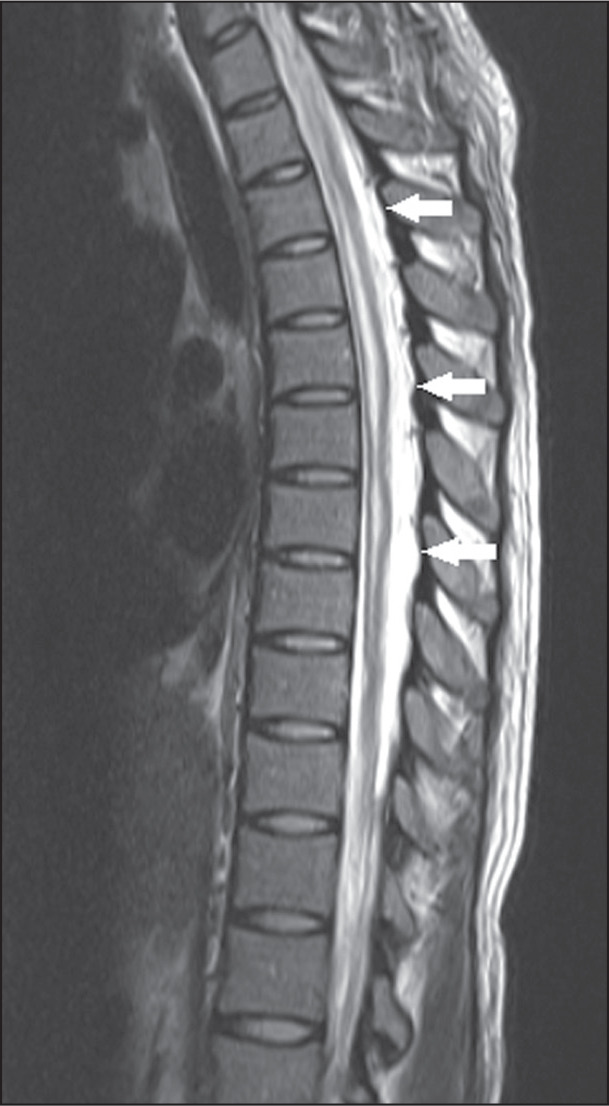
Zoster myelitis. Sagittal T2-weighted MRI sequence showing marked spinal atrophy together with a diffuse bilateral hyperintense signal (arrows).

### Zoster myelitis

Zoster myelitis is an infectious manifestation caused by reactivation of varicella-zoster virus that has remained inactive in the sensory ganglia since the first infection. Clinically, it is characterized by pain and rash. On MRI, the typical presentation is a hyperintense signal in the lateral portion of the spine on T2-weighted images, related to segments corresponding to the cutaneous eruption, without significant contrast enhancement, and it may evolve to segmental atrophy (Figure 10).

### Tuberculosis

**Figure 11:**
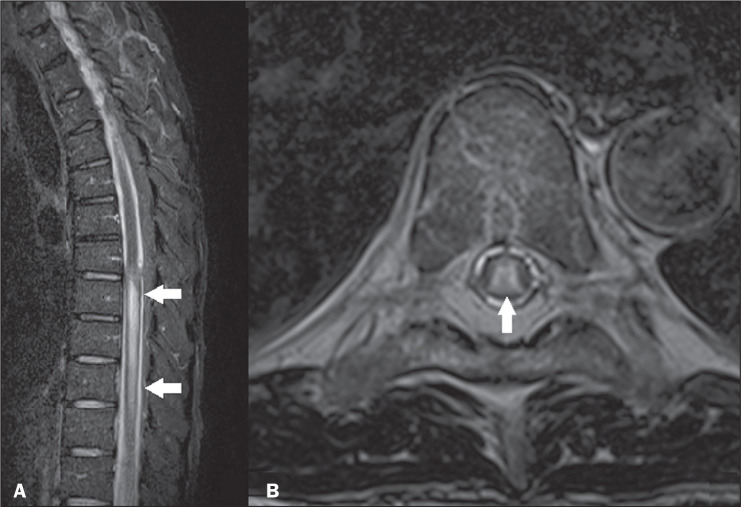
Tuberculosis. Sagittal STIR MRI sequence (A) showing a hyperintense signal in spinal cord lesions (arrows) and axial T2-weighted MRI sequence (B) showing a hyperintense signal in the central region of the spine (arrow).

Tuberculous myelitis is rare and, in some cases, evolves to ischemia and spinal cord necrosis. Spinal cord involvement may manifest as tuberculoma or transverse myelitis or can even be secondary to the involvement of structures adjacent to the spinal cord. The lumbar spine is the most common site of involvement, followed by the thoracic spine. On MRI, the most common pattern is one of a longitudinally extensive hyperintense lesion on T2-weighted images, more evident in the central region of the spine, in some cases occupying more than two thirds of its sectional area^**(15)**^, as depicted in Figure 11.

## CONCLUSION

In view of the aspects described above, it is obvious that spinal cord lesions pose a challenge for clinicians and radiologists. However, neuroimaging findings, when taken together with clinical and biochemical data, may facilitate the diagnosis and guide the treatment. Therefore, radiologists should be prepared to interpret such findings.
